# Supplementary material for: Structural basis for cofilin binding and actin filament disassembly
Source: Nat Commun. 2018 May 10;9:1860. doi: 10.1038/s41467-018-04290-w (PMC5945598; doi:10.1038/s41467-018-04290-w)
Supplement: Supplementary file 1 — Supplementary Information [file 41467_2018_4290_MOESM1_ESM.pdf]

Supplementary Information

Structural basis for cofilin binding and actin filament disassembly

Tanaka et al.

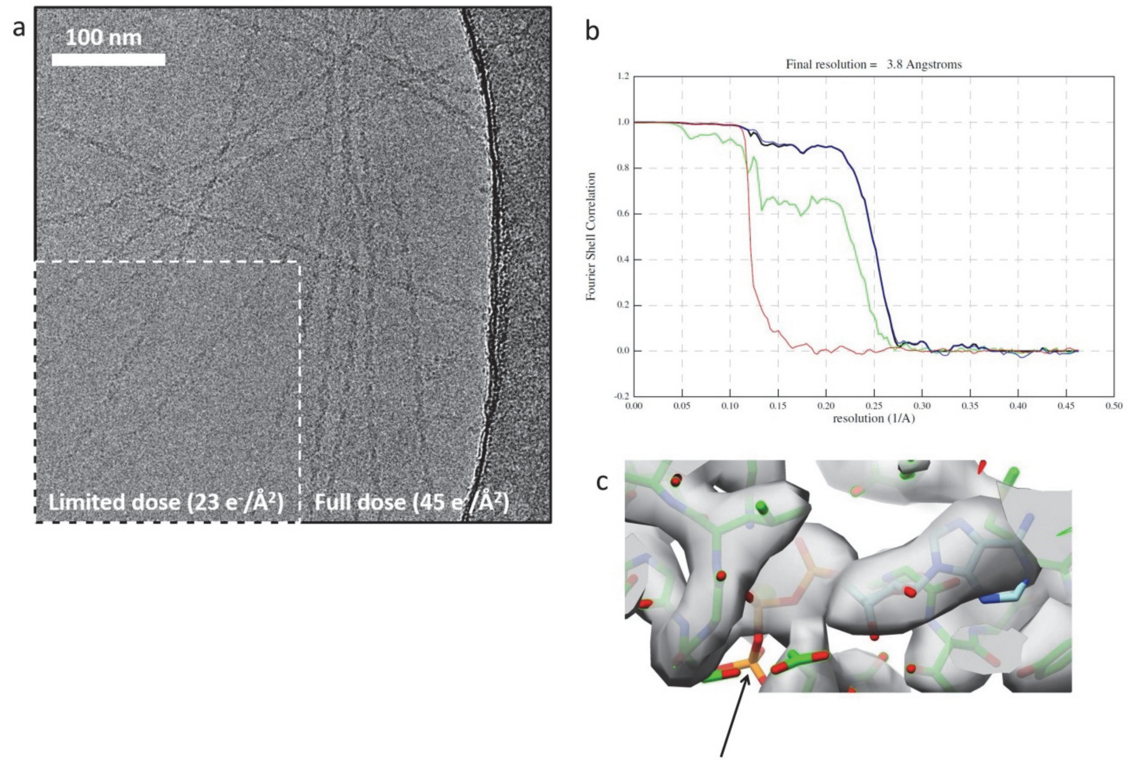

Supplementary Figure 1: An example of raw images and the evaluation of the map. a: An example of a cryo-electron microscope (cryo-EM) image. The inset enclosed by white dashed lines shows an image with a limited dose (25 $e^-/Å^2$), which was used for reconstruction, while the other part of the image is with a full dose (45 $e^-/Å^2$), which was used for boxing the filaments. **b:** Fourier shell correlations (FSCs) for evaluating the resolution calculated by RELION[1, 2]. FSCs between two 3D structures from each half of the dataset with or without masking are presented in green and blue, respectively. The red curve was calculated after the phase was randomized beyond 8.6 Å to evaluate the artifacts from overfitting[3]. The black curve is the corrected FSC after accounting for the artifacts from overfitting. Based on the golden standard criteria, the resolution is 3.8 Å with a threshold of 0.143. **c:** A cryo-EM map around the ADP from a different view to that in Fig. 1c. ADP in the cofilactin model was replaced by ATP. There was no density due to the γ-phosphate of the ATP (black arrow), indicating that the bound nucleotide in the map is ADP.

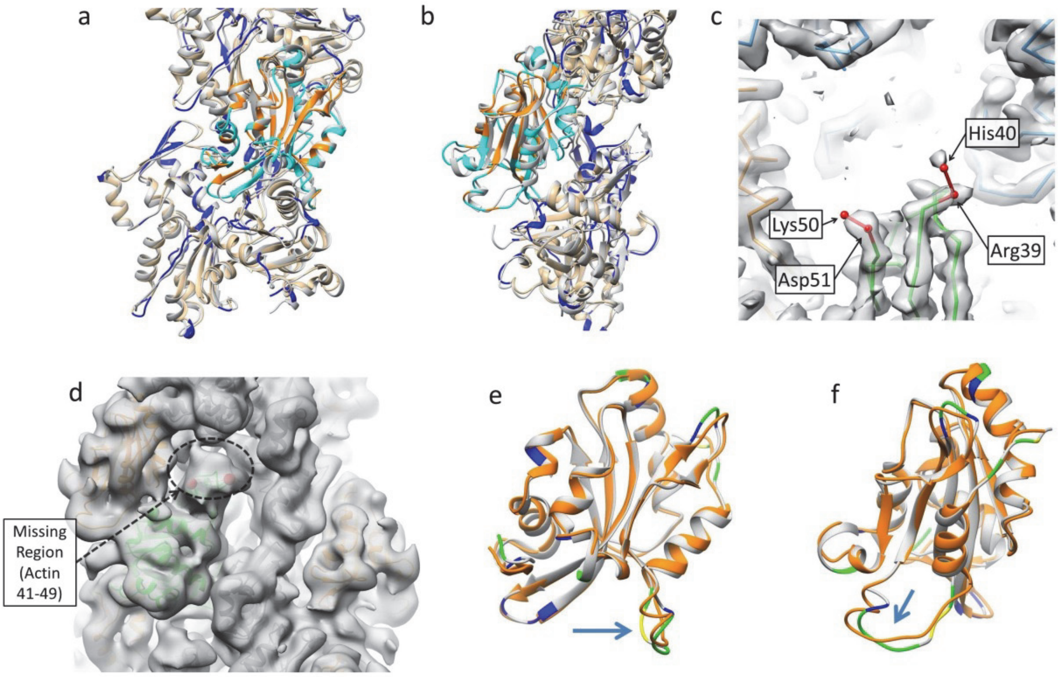

Supplementary Figure 2: Comparison with previous models. **a**: A comparison between the previous cryo-EM model at a 9 Å resolution[4] and the current model. Cofilin and the actin subunits in the current model are presented in orange and camel, respectively, and the previous model is presented in gray. The previous and current model structures were aligned, and the position difference of each Cα atom was calculated. The residues of actin and cofilin in the current model with a calculated position difference of over 1.5 Å are colored in blue and cyan, respectively. The root mean square distance (rmsd) of α-carbons between our cofilactin structure and the previous model is 1.6 Å. **b**: A 90° rotated view of **a**. **c**: A cryo-EM map around residues 40–50 of actin. The contour is 5.00 rmsd, which is lower than that of Fig. 1c, d and Supplementary Fig. 1c (6.00 rmsd). The α-carbons of the cofilactin model are superposed. No density was observed for actin 41–49, although the density for residues up to 39 and from 51 was obvious, indicating disorder of residues 41–49. **d**: A low-pass filtered map at 8 Å resolution. The main chains of the cofilactin model are superimposed. One actin subunit; the other actin subunits and cofilins are presented in green, gray and orange. The P-end is at the top. The position of one missing region (41–49 of actin) is indicated by a dotted ellipse, with the positions of residues 40 and 50 indicated by red spheres. The contour is 2.00 rmsd, which is much lower than that in the other figures. At this very low density contour in the low-pass filtered map, some densities emerge that appear to correspond to the missing residues. **e**: Comparison of cofilin in cofilactin (orange) with the human cofilin-1

crystal structure (4BEX, gray). The two cofilins were aligned to each other, and the position difference of each Cα atom was calculated. The residues in 4BEX with a calculated position difference of over 1.5 Å, 2 Å and 2.5 Å are colored in blue, green and yellow, respectively. The loop at residues 19–30 is indicated by a blue arrow. **f**: A 90° rotated view of **e**.

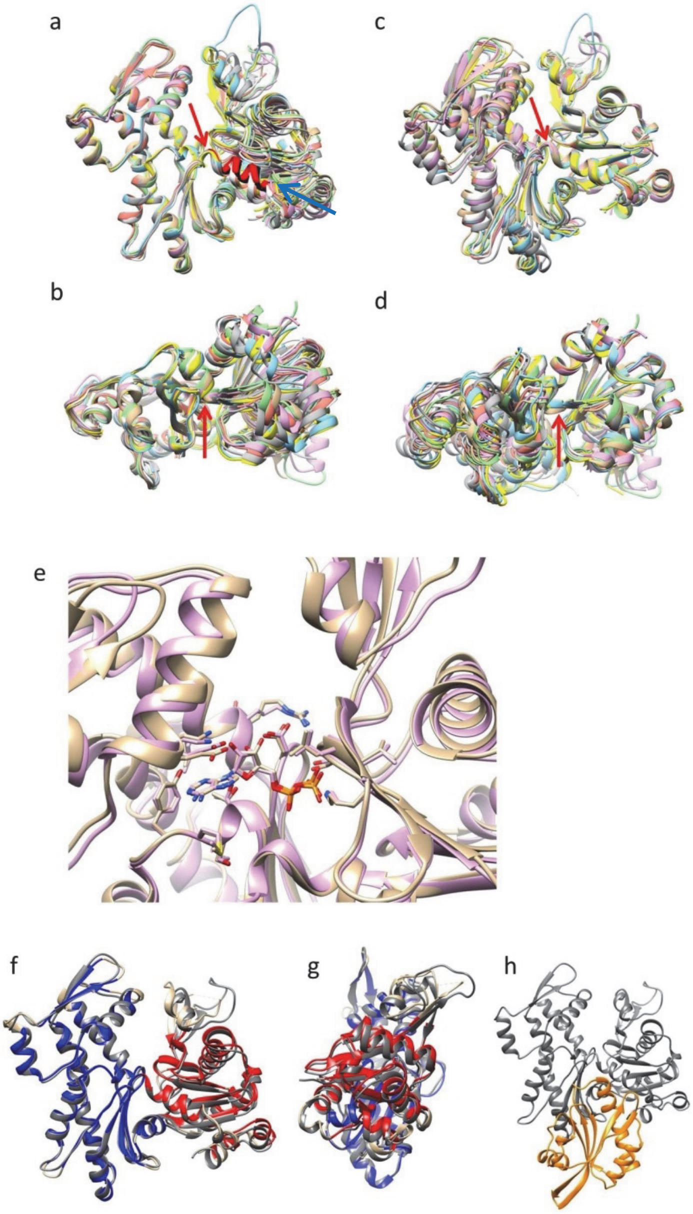

Supplementary Figure 3: Comparison with other actin structures. **a–d:** Structure comparison of eight actin structures: cofilactin in camel, G-actin (1J6Z) in magenta, F-actin (5JLF) in cyan, actin in actin-twinfilinC (3DAW) in light green, actin in the actin-gelsolin1-tropomodulin actin-binding domain complex (4PKH) in orange, F-form actin from a recently obtained crystal structure (Takeda et al., unpublished results) in yellow, actin in the actin-profilin-VASP peptide complex (2PAV) in gray, and the actin portion of actin-thymosin beta 4 hybrid protein (4PL7) in white. **a:** The structures were superposed by aligning the ID rigid bodies (blue in Fig. 2I). One α-helix (337–346) of the cofilactin and the actin-twinfilinC complex is colored in red or black, respectively, and

indicated by a blue arrow. The α-helix position in the two structures is distinct from the other models, indicating that the relative orientation of the two rigid bodies of the two structures is distinct from the others. **b**: Bottom view of **a**. **c**: The structures were superimposed by aligning the SD1 rigid bodies (red in Fig. 2i). **d**: Bottom view of **c**. The hinges between the two rigid bodies are indicated by red arrows, one at residue 336 (**a** and **c**) and one at residue 137 (**b** and **d**). **e**: The structure around the ADP of G-actin (1J6Z) and the actin within cofilactin are in magenta and camel, respectively. The two structures were superposed by aligning the α-carbons of the residues around the ADP, 13–16, 156–158, 182–185, 213–214 and 301–306; the side chains of these residues are represented by sticks. **f**: Structure comparison between the actin in cofilactin and in the actin-twinfilinC complex (3DAW, in gray). The rigid bodies of the ID and SD1 and the remainder of the actin molecule in cofilactin are colored in red, blue and camel, respectively. The rigid bodies of the IDs were aligned with each other. The orientation of the rigid bodies of SD1 in the two structures is almost identical. **g**: A 90° rotated view of **f**. **h**: The entire structure of the actin-twinfilinC complex (3DAW). TwinfilinC (orange) binds the actin monomer (gray) through the G-site.

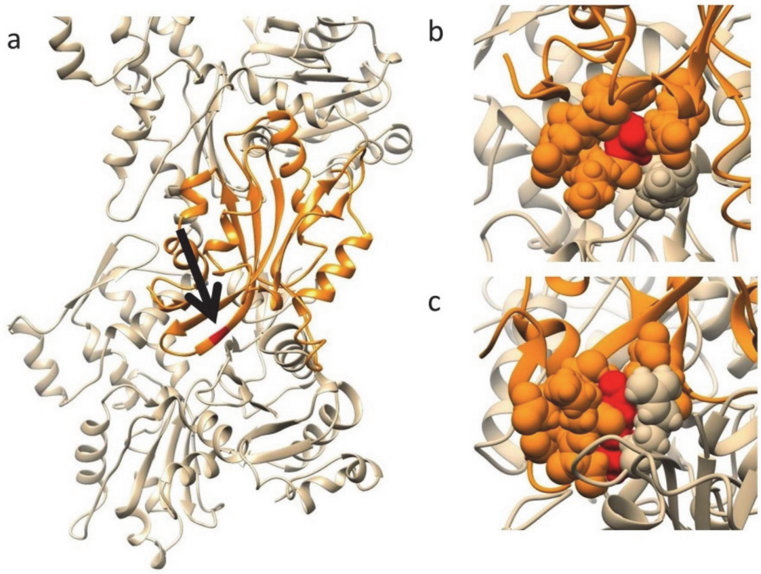

Supplementary Figure 4: Structure around K96 of cofilin. a: Overview. K96 of cofilin in cofilactin is colored in red and indicated by a black arrow. **b:** K96 of cofilin and the surrounding side chains are visualized as space-filling models. Cofilin residue K96, cofilin and the actin subunit are colored in red, orange and camel, respectively. **c:** A rotated view of **b**.

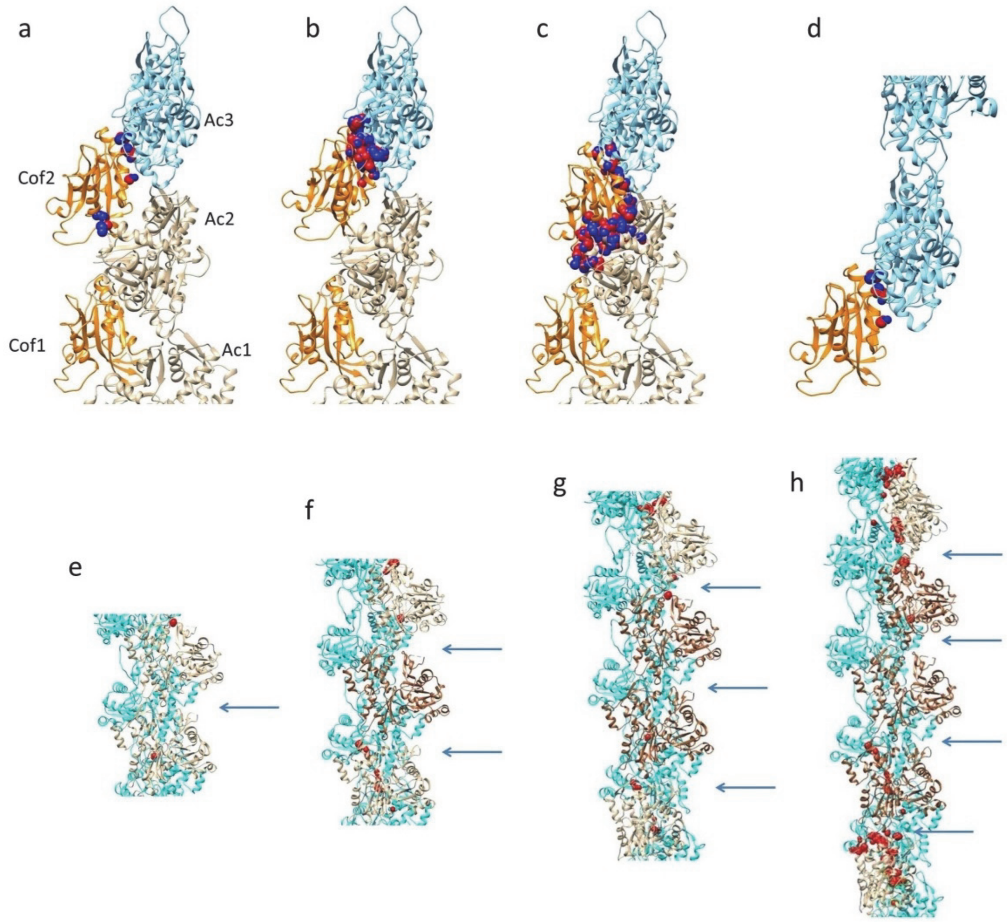

Supplementary Figure 5: Expected collisions between molecules. a: The collisions expected when the second cofilin (Cof2) binds to the P-end side of Cof1, which is already bound to actin subunits Ac1 and Ac2 (both in camel). The upper actin subunit, Ac3, remains in the F-form (cyan). A model was constructed for Cof2 bound to the Gi-site of Ac3. Atoms that are expected to collide between actin subunits (Ac2, Ac3) and cofilin (Cof2) are shown in space-filling representation in blue and red, respectively. **b:** Collisions expected when Cof2 binds to the F-site of Ac2. **c:** Collisions expected when Cof2 binds to the Go-site of Ac3. **d:** Collisions expected by cofilin (orange) binding to the Gi-site of the B-end subunit of the actin filament (cyan). **e-h:** Collisions expected between the two strands when cofilin molecules bind to one of the two strands of the actin filament. Actin subunits are color coded, depending on the number of interacting cofilin molecules: none in cyan, one in camel and two in brown. Bound cofilin molecules are not displayed for clarity. $\Delta\phi$ of the cofilin-bound strand (brown and camel) and unbound strand (cyan) is 35.8° and 26.8°, respectively. The colliding atoms are shown in red space-filling representation. Blue arrows indicate the cofilin binding positions. One, two, three and four cofilins are bound to the actin filament in **e**, **f**, **g** and **h**, respectively.

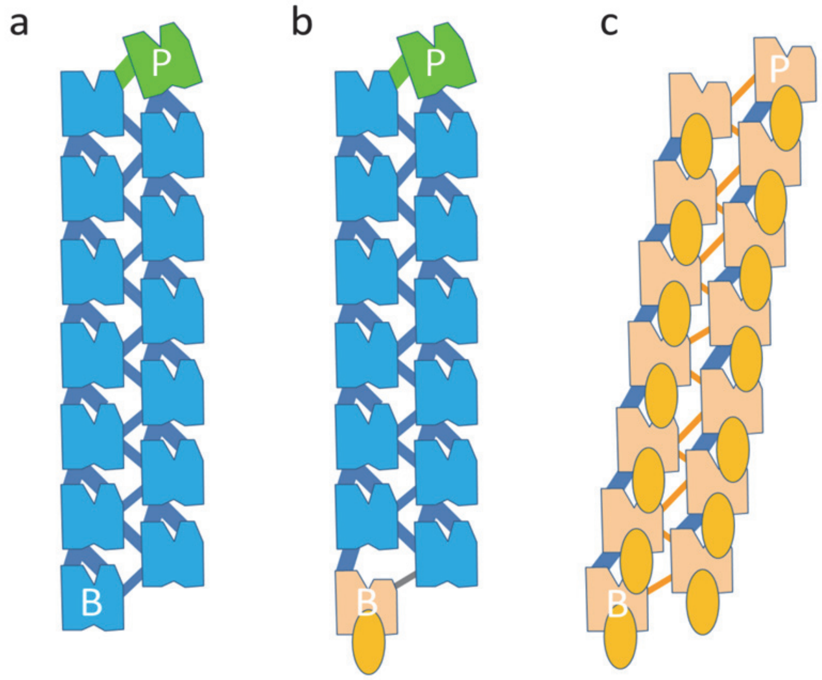

Supplementary Figure 6: Models of the effects on depolymerization. a: Schematic illustration of the actin filament with the P-end and B-end subunits labeled with white letters. The P-end subunit (green) is tilted towards the opposite strand, forming a specific contact (green line). **b:** One cofilin binds to the B-end subunit. The binding alters the B-end subunit into the C-form, and the intrastrand OD-ID interaction is disrupted. **c:** A fully cofilin-decorated actin filament.

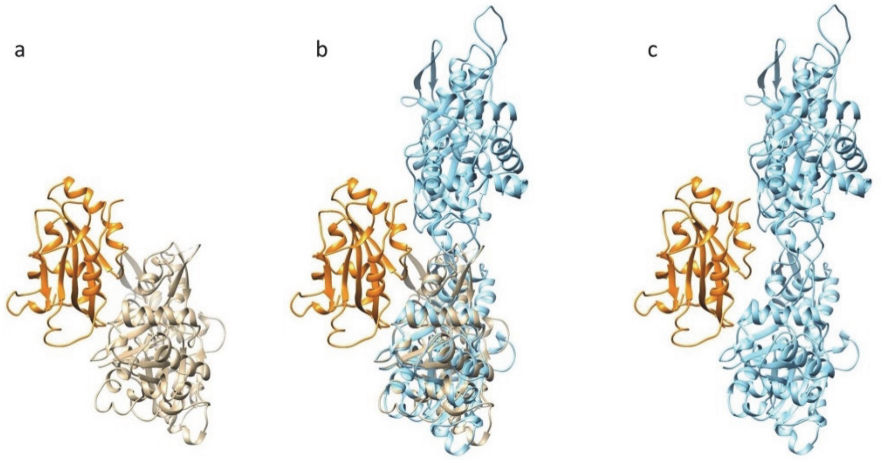

Supplementary Figure 7: Constructing models of cofilin bound to the F-site of the F-actin structure. **a**: One actin subunit (camel) and one cofilin (orange) bound to the actin subunit through the F-site in cofilactin. **b**: The SD1 rigid body of the actin subunit in **a** was aligned against the SD1 of one of the F-actin subunits (cyan). **c**: Cofilin in **b** was combined with F-actin.

Supplementary Table 1: Residues in the actin-cofilin interface

F-site on actin	26–28,57,91–93,95–97,100,336–337
F-site on cofilin	18–23,86,93–99,130,132
Gi-site on actin	140, 143–149, 291–292, 296, 326, 334
Go-site on actin	341–342, 345–346, 348–351, 355
G (Go+Gi)-site on cofilin	3–5, 45, 103, 105, 107, 111–112, 114–116, 118–119, 121, 136, 138, 142, 151–152
Gi_l-site	Ac291–Cof151, Ac292–Cof152, Ac326–Cof142, Ac296–Cof136

Supplementary Table 2: Residues constituting the rigid bodies

Inner domain	138–145, 147–166, 171–194, 200, 205–230, 236–242, 246–322, 326–336
Subdomain 1	7–35, 68–71, 76–108, 115–137, 337–347, 355–364, 369-371

Supplementary Table 3: Cryo-EM data collection, refinement and validation statistics

	#1 name<br>(EMDB-6844)<br>(PDB 5YU8)
**Data collection and processing**	
Magnification	75 k
Voltage (kV)	300 kV
Electron exposure (e–/Å^2)	24
Defocus range (μm)	1~3
Pixel size (Å)	1.1
Symmetry imposed	Helical
Initial particle images (no.)	202,217
Final particle images (no.)	86,388
Map resolution (Å)<br>FSC threshold	3.8<br>0.143
Map resolution range (Å)	∞ ~ 3.8
**Refinement**	
Initial model used (PDB code)	1J6Z, 1TVJ, 2ZWH
Model resolution (Å)<br>FSC threshold	3.8<br>0.143
Model resolution range (Å)	∞ ~ 3.8
Map sharpening *B* factor (Å^2)	-138.4
Model composition<br>Non-hydrogen atoms<br>Protein residues<br>Ligands	 18155<br>2297<br>10
B factors (Å^2)<br>Protein<br>Ligand	 -<br>-
R.m.s. deviations<br>Bond lengths (Å)<br>Bond angles (°)	 0.015<br>1.950
Validation<br>MolProbity score<br>Clashscore<br>Poor rotamers (%)	 0.75<br>0.3<br>0.25
Ramachandran plot<br>Favored (%)<br>Allowed (%)<br>Disallowed (%)	 97.2<br>2.6<br>0.2

Supplementary References

1. He S, Scheres SHW. Helical reconstruction in RELION. *Journal of structural biology* **198**, 163-176 (2017).

2. Scheres SH. RELION: implementation of a Bayesian approach to cryo-EM structure determination. *Journal of structural biology* **180**, 519-530 (2012).

3. Chen S*, et al.* High-resolution noise substitution to measure overfitting and validate resolution in 3D structure determination by single particle electron cryomicroscopy. *Ultramicroscopy* **135**, 24-35 (2013).

4. Galkin VE*, et al.* Remodeling of actin filaments by ADF/cofilin proteins. *Proceedings of the National Academy of Sciences of the United States of America* **108**, 20568-20572 (2011).
