# Supplementary material for: Structural basis for cofilin binding and actin filament disassembly
Source: Nat Commun. 2018 May 10;9:1860. doi: 10.1038/s41467-018-04290-w (PMC5945598; doi:10.1038/s41467-018-04290-w)
Supplement: Supplementary file 3 — Description of Additional Supplementary Files [file 41467_2018_4290_MOESM3_ESM.pdf]

Description of Additional Supplementary Files

File Name: Supplementary Movie 1
Description: Transition between the F- and G-forms. Transparent ribbon models in cyan and magenta are F-from (5JLF) and G-form (1J6Z), respectively. The ID and SD1 rigid bodies are presented in blue and red, respectively.

File Name: Supplementary Movie 2
Description: 90° rotated view of Supplementary Movie 1.

File Name: Supplementary Movie 3
Description: Transition between the C- and G-forms. Transparent ribbon models in green and magenta are C-from and G-form (1J6Z), respectively. The ID and SD1 rigid bodies are presented in blue and red, respectively.

File Name: Supplementary Movie 4
Description: 90° rotated view of Supplementary Movie 3.

File Name: Supplementary Movie 5
Description: Movie version of Fig. 3d.

File Name: Supplementary Movie 6
Description: Movie version of Fig. 3e.

File Name: Supplementary Movie 7
Description: Actin-Cofilin interactions. Actin subunits are shown as a space-filling model. Cofilin is presented in orange. The F-site (Cofilin-B-subunit interface), Go site (Cofilin-SD1 of the P-subunit interface), Gi_l site (Cofilin-ID of the P-subunit interface with long-range interactions) and Gi_s-sites (Cofilin-ID of the P-subunit interface which is not included in the Gi_l site) in the actin subunits are shown in blue, red, brown and yellow, respectively. In cofilin, residues forming F- and G-sites are represented in blue and green, respectively. The cofilin residues contributing to the binding sites are shown in a stick model.

File Name: Supplementary Movie 8
Description: Model of cofilin binding. Actin subunits in the cofilactin structure, the F-actin structure and an intermediate structure are presented in camel, cyan and magenta, respectively. Cofilin is presented in orange. The binding interface is colored in the same manner as in Figs. 4a–f and 5.

File Name: Supplementary Movie 9
Description: 90° rotated view of Supplementary Movie 8.
